# Ten-year longitudinal study of factors influencing nocturnal asthma symptoms among Asian patients in primary care

**DOI:** 10.1038/npjpcrm.2015.64

**Published:** 2015-10-29

**Authors:** Ngiap Chuan Tan, Nivedita V Nadkarni, Weng Kit Lye, Usha Sankari, van Hai Nguyen

**Affiliations:** 1 Department of Research, SingHealth Polyclinics, Singapore, Singapore; 2 Centre of Quantitative Medicine, Duke-NUS Graduate Medical School, Singapore, Singapore; 3 Health Services and Systems Research Program, Duke-NUS Graduate Medical School, Singapore, Singapore

## Abstract

**Background:**

Nocturnal asthma symptoms result in poor quality of life and morbidity.

**Aims:**

This study primarily examines key factors predicting and mitigating nocturnal symptom risks among asthma patients, who were enrolled into a Singapore publicly funded asthma care programme. It also studies the association between nocturnal symptoms and medication changes as the secondary outcome.

**Methods:**

A longitudinal study was conducted on 939 multi-racial Asian patients with persistent asthma. Patient clinical and therapeutic data were retrieved retrospectively from the programme’s database established in 2004. Association between nocturnal symptoms (defined as night-time cough, wheeze and breathlessness at least twice monthly) and each categorical predictor was tested. The generalised linear mixed-effects model (GLIMM) was used to model the primary and secondary outcomes.

**Results:**

Having nocturnal asthma symptoms was significantly associated with the number of days with breathlessness, off usual activities and off work, and asthma severity at baseline (all *P* values <0.05). The asthma action plan (AAP) status is significantly associated with nocturnal symptoms after adjusting for race, age and smoking status at baseline (odds ratio (OR)=0.49 (updated asthma action plan versus none), OR=0.37 (been-on plan versus none)). The risks of nocturnal asthma symptoms increased over time for those with allergic rhinitis (OR=1.52) and reduced with subsequent visits (OR=0.91). Nocturnal symptoms increased the odds (OR=2.87) of switching from inhaled corticosteroid (ICS) to combination medications (ICS-LABA (long-acting β_2_-agonist)).

**Conclusions:**

The risks of having nocturnal symptoms were primarily associated with those with allergic rhinitis. An increase in patients’ nocturnal symptoms was also predictive of the switching from ICS medications to combination formulations by their physicians.

## Introduction

Globally asthma affects an estimated 300 million people.^1^ Approximately 47 to 75% of them report nocturnal symptoms.^2^ Nocturnal asthma is defined as night-time worsening of reversible airway disease associated with an increase in symptoms and airway responsiveness.^3^ Those affected by nocturnal asthma tend to cough, wheeze, have dyspnoea, be awakened at night, leading to high morbidity. However, the use of inhaled corticosteroid (ICS as controller medication) and long-acting β_2_-agonist (LABA) have been shown to reduce their night-time symptoms and improve their psychometric outcomes, and quality of life.^4^ In the same review by William Calhoun, he also indicated that allergen exposure could also aggravate nocturnal asthma symptoms.^4^ Understanding and addressing these factors are prerequisites to the design of targeted interventions. Identifying patients affected by nocturnal asthma becomes a priority, as appropriate measures can be implemented to reduce their night-time symptoms. Nocturnal asthma leads to a diurnal reduction in forced expiratory volume in one second (FEV_1_) of more than 15%.^5^ However, access to diurnal spirometry data for the diagnosis and management of nocturnal asthma is limited in many developing and even in some developed countries.^6,7^ Nocturnal asthma is thus often gauged by the presence of nocturnal asthma symptoms, which are probed by attending physicians or other health-care professionals, self-reported, or are incorporated as part of multi-faceted questionnaires to assess asthma control, such as the Asthma Control Test.^8,9^

Asthma is also common in Singapore, affecting 10.5% of the adult population aged 18-69 years.^10^ It is largely managed in the primary health-care system, served by dual providers in the public polyclinics and private general practitioner clinics. The National Health Survey in 2010 showed that 52% of local asthma patients had one episode of asthma symptoms in the 12 months before the survey, with 30% reporting symptoms that disturbed their sleep.^10^ Few local studies focused specifically on patients’ nocturnal asthma symptoms. A primary care study by Tan NC *et al*.^11^ found that adult patients with asthma who smoked had increased nocturnal symptoms. Another tertiary-based study showed that markers of asthma severity, Malay and Indian race, smoking, discontinuity of care and self-care behaviour were risk factors for acute resource utilisation.^12^ The long-term use of known effective medications, such as ICS, and LABA, can be costly in the fee-for-service primary health-care system in Singapore. Medication cost becomes a hindrance towards the selection of appropriate medication and maintenance of its continuous use to control asthma.^13^ The Singapore National Asthma Programme was implemented in 2003 to address these multifactorial causes of morbidities among local patients with asthma.^14^ Sponsored by governmental ‘Reinvestment Fund’ (RF), this asthma care programme provided an opportunity to undertake a decade-long study of a cohort of asthma patients in primary care.

The primary objective of this longitudinal study is to identify and quantify the risk factors associated with an increase or a reduction of nocturnal asthma symptoms over time in Singapore’s public primary care setting. The primary hypothesis is that risk factors such as demographic characteristics of patients and trigger factors are associated with the persistence of nocturnal symptoms over time. The secondary hypothesis is that change in inhaled asthma medication usage over time is associated with the frequency of nocturnal symptoms.

## Materials and methods

### Data source

The RF asthma care programme database was established in 2003 to record the enrolled patients’ demographic characteristics, asthma severity based on Global Initiative for Asthma (GINA) classification (2006), exacerbation information (include frequency of rescue therapy at designated polyclinic and those which occurred by other provider such as the emergency unit in hospital), medical consultation, asthma-related hospitalisation, type and dose of asthma medications and treatment expenditures.^14^ These data are serially recorded on standardized data forms during each patient visit by the asthma team at the respective polyclinic. Certain information such as patients’ self-report of asthma trigger factors and associated allergic syndromes such as allergic rhinitis, eczema, allergic conjunctivitis were recorded only once at enrolment.

### Study population

The study population comprises patients who were enrolled into this programme at different times during the period 2004–2013. They are managed at two selected SingHealth (SHP) polyclinics in the districts of Outram and Pasir Ris, respectively.^15^

The patients include both gender, all age groups, multi-racial composition (Chinese, Malays, Indians and Others) with physician-diagnosed asthma. They had suboptimal asthma control at enrolment, defined as (1) two or more acute care contacts (primary care or emergency unit in hospitals for acute rescue therapy) within a month; (2) poor asthma control requiring the use of reliever inhaler three or more times per week; (3) past history of severe exacerbation requiring intubation or intensive care; (4) asthma-related hospitalisation in the preceding 3 months; (5) other risk factors such as poor home support and poor adherence to treatment. Another group of enrolled patients included those who were referred to the designated polyclinics for continuity of care after their asthma control was stabilized at pulmonologist clinics in local public hospitals.

At each visit to the designated site, patients are reviewed by an attending polyclinic physician, who may review and update their written asthma action plan (AAP) periodically, especially when there is a change in their medication regime. They are then counselled by the asthma-trained nurse, who reinforces their personal AAP, and provides them with the next review date. The nurses monitor their scheduled visits using the polyclinic patient information system and contact defaulters to arrange for alternative visits. Patients can seek consultations on unscheduled visits when they are symptomatic.

Patients have the option of visiting other health-care providers, including hospital emergency units, outside the polyclinic operating hours. Data from these off-site asthma consultations are also recorded in the database via self-reporting from patients during subsequent consultations at designated polyclinics.

### Variables of interest

The primary outcome variable is the presence or the absence of nocturnal asthma symptoms, defined as night-time cough, wheeze and breathlessness at least twice monthly. The secondary outcome is the change from controller medication to combined ICS and LABA medication or vice versa between clinic visits. The primary predictors of interest postulated to affect the presence of nocturnal symptoms are the socio-demographic variables of age, gender and race and associated allergic syndromes.

### Statistical analysis

The analysis focuses on assessing the association between the response and the predictors of interest. Continuous predictors have been tabulated by mean (standard deviation) or median (interquartile range) and categorical predictors by frequency *N* (%). Associations between the primary outcome, secondary outcomes and other categorical predictors of interest were tested using the Chi-square test or Fisher’s exact test as appropriate. The primary outcome at baseline was analysed using univariate and multivariable logistic regression.

Longitudinal data analysis was performed using the generalised linear mixed-effects (GLIMM) model for the primary outcome adjusting for visit within subject and the site as independent random effects and other covariates as fixed effects. The GLIMM approach was used to analyse the secondary outcome adjusting for subject as random effect and other covariates as fixed effects.

The mixed-effects model allows all non-missing data to be used in the analysis without imputation. However, for the estimate of the parameter to be unbiased, it is required that the distribution of the missing data, conditional on the variables in the model and the observed data, does not depend on the values of the missing data, so that the data are missing at random. Even though the mixed model approach is able to handle missing data in the estimation procedure under the missing-at-random assumption, the number of observations at each visit should not fall under half of the observations at the first visit to ensure robust estimation of the effect and the corresponding standard error.^16,17^ Therefore, although the number of visits ranges from 1 to 82 visits, the data were analysed taking the first 10 visits of each patient (including the baseline visit) into account, because the total number of observations after the first 10 visits was less than half the number at baseline.

The independent variables used for the analysis at baseline were different from some of the variables in the longitudinal analysis because most of the information was collected at baseline. Variables such as smoking status for example are time dependent and therefore could not be analysed longitudinally. Only time-independent variables like race, gender, etc. were analysed longitudinally. We also assumed that triggers like eczema, rhinitis, conjunctivitis and smoke were time independent.

The secondary outcome was analysed using GLIMM adjusting for subject as a random effect and other covariates as fixed effects. For this analysis, only those visits where there was a change in medication use were selected. This resulted in a different data set from the one used for the analysis of the primary outcome. This placed the emphasis on how nocturnal symptoms impacted change in medication. All the statistical analyses were done in R version 3.0.0 and SAS version 9.3.

This study was screened by SingHealth Centralized Institution Review Board (CIRB no: 2014/167/E), which exempted the study from ethical review, as de-identified data were retrieved retrospectively from the RF database.

## Results

**Figure 1:**
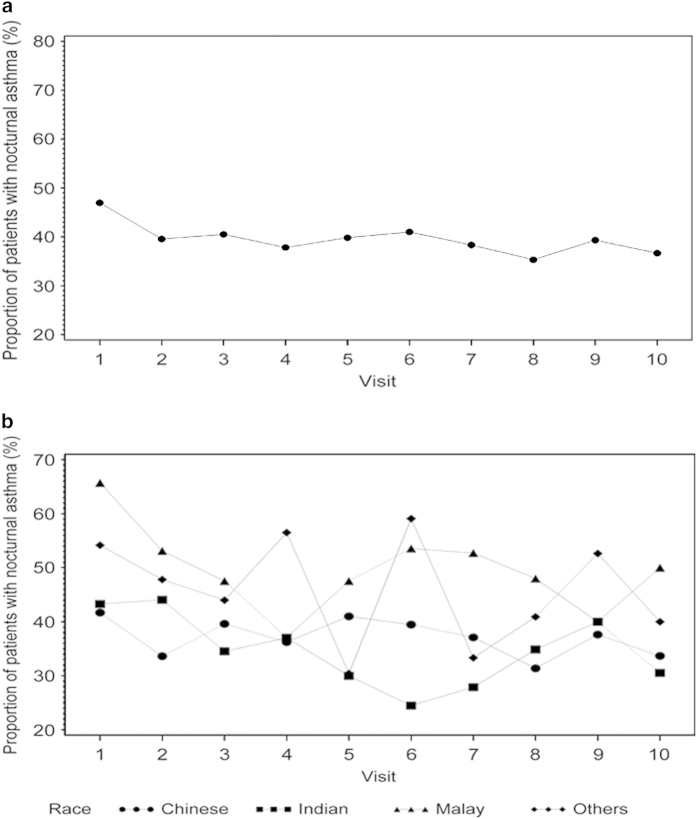
(**a**) Proportion of patients with nocturnal asthma (%). (**b**) Proportion of patients with nocturnal asthma (%) by race.

The baseline characteristics of the 939 enrolled patients are tabulated by the primary outcome in Table 1. Figure 1 shows the proportion of subjects with nocturnal symptoms at each visit who were analysed at baseline and at subsequent visits in the longitudinal analysis. Five hundred and fifty-four (59%) patients had nocturnal symptoms at recruitment. Of these, 16.7% patients had at least 2 or more days off work per month, 91.2% had day symptoms and 31.1% had allergic rhinitis. Their mean age was 44.8 years and comprised males (45.5%), Chinese (57.4%), Malay (13.7%), Indian (22%) and others (6.9%). Having nocturnal symptoms was significantly associated with the status of the AAP, number of days with breathlessness, off usual activities and off work (all *P* values <0.05) at baseline. The status of the written AAP was predictive of nocturnal symptoms at baseline both in the univariate regression as well as in the multivariable regression. Results for the univariate and multivariable analyses are tabulated in Table 2. Those who were not on the AAP at baseline had a higher proportion of having nocturnal symptoms compared with those who were either on the plan or had updated it. The multivariable analysis at baseline tabulated in Table 2 suggests that either being on the AAP or updating it was instrumental in assisting in the control of nocturnal asthma symptoms at baseline (odds ratio (OR)=0.49 (been-on plan versus none), OR=0.37 (updated versus none), *P* values for both <0.001).

The longitudinal multivariable modelling indicates that the odds of having nocturnal symptoms increases for patients having allergic rhinitis (OR=1.52) and decreases marginally with age (OR=0.99) and on each subsequent visit (OR=0.91) conditional on the random effects. Results are tabulated in Table 3. The longitudinal modelling on the secondary outcome was performed on a subset of those patients who experienced a change in medication over different visits: it suggests that nocturnal symptoms are strongly predictive of a recent change from single controller medication to combination medication (OR=2.87) and the likelihood of change in medication marginally increases with age (OR=1.01), conditional on the random effect over time (Table 4).

## Discussion

### Main findings

Our primary hypothesis was to identify key predictors for the occurrence of nocturnal symptoms over time. Patients with allergic rhinitis had significantly increased odds of having nocturnal symptoms. Allergic rhinitis has been strongly associated with suboptimal asthma control due to the link between diseases of the upper and lower airways.^18^ Postnasal drip secondary to allergic rhinitis will aggravate cough and affect breathing when asthma patients lie supine during sleep at night. Persistent allergic rhinitis is prevalent in tropical Singapore.^19^ Although it is difficult to eradicate the triggers and the underlying immunological pathology of allergic rhinitis, it can be adequately controlled with medications. Treatment of allergic rhinitis is thus a modifiable factor to reduce nocturnal asthma symptoms.^18^ However, patients expect quick relief with low-cost medications such as antihistamine for this chronic disease, instead of the more costly nasal steroid.^19^ As a result, the suboptimal treatment of allergic rhinitis can lead to persistent nocturnal asthma symptoms. A concerted effort among local primary care physicians is needed to educate their patients and steer them towards accepting evidence-based treatment for both asthma and allergic rhinitis. The local national health financing system has recently extended subsidised care for patients receiving treatment at private general practitioner clinics (the Community Health Assist Scheme), which is timely to address the cost barrier and enables patients to receive appropriate drug treatment for allergic rhinitis at acceptable costs.^20^

Age seemed to decrease the odds of having nocturnal symptoms over time. It may be associated with adult patients having higher capability and capacity for self-management and use of AAP owing to repeated counselling by the primary health-care team. These postulations need verification with further research.

We adjusted for potential confounders such as site (Outram, Pasir Ris) in the regression analysis. It is known that the proportion of different racial groups is different in Pasir Ris compared with Outram in the RF database. Therefore, the variables site and race are confounded with each other. As a result, when site was included as a random effect in the model, race was no longer significant. Race has previously been postulated as being clinically relevant and was therefore retained in the model.^21^

Referring to the secondary hypothesis, the odds of change in medication from controller to combination formulation were three times more for someone with night symptoms. Physicians could have leveraged on the history of nocturnal symptoms among patients as a decision point to titrate their medication. This conforms to guidelines as a therapeutic intervention to re-establish asthma control.^5^ The cost barrier towards the use of the more costly medications is lowered owing to the subsidised medications under the RF asthma care programme, thus allowing the patients to access the appropriate medication compatible with their asthma status.

As expected, nocturnal symptoms were highly associated with asthma severity, days off work, days off regular activities, and days off owing to breathlessness. What was significant was its association with the status of patients’ asthma action plans. Having an updated AAP reflects that the patient was reviewed by the polyclinic health-care team. The AAP also educates the patient to make short-term changes to their treatment in responses to their symptoms, including those occurring at night.^21^ Gibson PG *et al*.^21^ had reported that adults seemed to benefit more from self-management education, with reference to their personal AAP, especially if the AAPs include both a step-up in inhaled corticosteroid doses and the addition of short course of oral steroid. Such measures are integral components of the AAP provided at the polyclinics.

### Strengths and limitations of this study

This longitudinal study provides an unprecedented insight into the asthma disease burden in a developed Asian community over time. Using longitudinal data analysis and the GLIMM modelling approach, it allows an appreciation of the evolving asthma status of the cohort of patients in a changing primary care environment, where new drugs and health-care policies are introduced into the health-care system periodically.

The truncation of the data on number of visits at 10 visits might introduce bias in the estimation. However, this is necessary for the unbalanced number of visits owing to staggered entry of patients into the programme. When we conducted a sensitivity analysis in which data on the entire range of visits were used, the estimates indicated that the standard errors were underestimated.

Retrospective data extraction from the database has its inherent limitations, such as missing or incongruent variables. Audit checks on the data set are carried out regularly as the data are submitted to the Ministry of Health every month. An additional round of quality check and rectification was performed by US, the co-author (the institution data management officer) before the de-identified data were submitted to the biostatisticians in the team.

### Interpretation of findings in relation to previously published work

Nocturnal asthma remains prevalent, even in developed nations with established primary health-care system. The prevalence rate of 59% in this study is similar to that in France (60%).^2^ The results support the recommendations by asthma clinical practice guidelines to introduce asthma action plans and evidence-based effective medications to patients.^5^ Although the implementation of some these measures has led to a decline in the prevalence of nocturnal asthma among the patients in the RF programme, about a third of them continue to have nocturnal symptoms. Efforts should be targeted at the other modifiable factors to further reduce the magnitude of this morbidity, such as reviewing and instituting appropriate level of treatment for allergic rhinitis and introducing measures to curb cigarette smoking.

### Implications for future research, policy and practice

Within a fee-for-medical service Asian community, the cost of asthma medications, especially the more expensive ICS-LABA combination medications, exerts a significant influence on the prescribing habits of the primary care physicians.^21^ As the RF programme mitigates this cost barrier to ensure equitable access to effective medications, our results imply that these physicians can potentially be influenced to change their choice of medications by probing into the magnitude of the nocturnal asthma symptoms of their patients. Physicians’ efficiency in their clinical assessment of disease status and decision-making mechanism in the choice of drug treatment for their patients in a time-sensitive and cost-conscious practice setting should be explored with further research.

### Conclusions

Nocturnal asthma was prevalent, especially affecting those with allergic rhinitis. Nocturnal symptoms were mitigated among patients who were older, whose asthma action plans and formulations of medications were reviewed and adjusted by their attending physicians.

## Figures and Tables

**Table 1:** Baseline characteristics of the asthma study cohort

	*Overall (*N*=939)*	*No night symptom (*N*=385)*	*Night symptom (*N*=554)*
Age	46.6 (23.0)	48.6 (23.1)	45.3 (22.8)
Duration of asthma	17.5 (16.8)	17.9 (16.7)	17.2 (16.9)
Weight	60.4 (20.8)	61.3 (19.8)	59.8 (21.4)
Height	152.0 (23.1)	152.6 (21.3)	151.7 (24.3)
Gender (Male)	418 (44.5)	166 (43.1)	252 (45.5)

*Race*			
Chinese	564 (60.1)	246 (63.9)	318 (57.4)
Malay	132 (14.1)	68 (17.7)	122 (22.0)
Indian	190 (20.2)	56 (14.6)	76 (13.7)
Others	53 (5.6)	15 (3.9)	38 (6.9)
Rhinitis (Yes)	288 (30.7)	116 (30.1)	172 (31.1)
Conjunctivitis (Yes)	29 (3.1)	14 (3.6)	15 (2.7)
Eczema (Yes)	79 (8.4)	28 (7.3)	51 (59.0)

*Cigarette smoking*			
Current	64 (6.8)	20 (5.2)	44 (7.9)
Ex-Smoker	104 (11.1)	40 (10.4)	64 (11.6)
Never	771 (82.1)	325 (84.4)	446 (80.5)

*Smoke as a trigger factor*			
Yes	373 (39.8)	157 (40.8)	216 (39.1)
No	549 (58.6)	222 (57.7)	327 (59.2)
Unknown	15 (1.6)	6 (1.6)	9 (1.6)

*Day off work*			
⩽2/month	94 (11.2)	21 (6.0)	73 (14.9)
⩽2/week	4 (0.5)	0 (0.0)	4 (0.8)
⩾3/week	7 (0.8)	2 (0.6)	5 (1.0)
None	735 (87.5)	327 (93.4)	408 (83.3)

*Day off SOB*			
⩽2/month	201 (21.5)	48 (12.6)	153 (27.6)
⩽2/week	177 (18.9)	44 (11.5)	133 (24.0)
⩾3/week	252 (26.9)	38 (10.0)	214 (38.6)
None	306 (32.7)	252 (66.0)	54 (9.8)

*Usual activities (number of days off work or school)*			
⩽2/month	47 (5.3)	7 (1.9)	40 (7.7)
⩽2/week	8 (0.9)	1 (0.3)	7 (1.3)
⩾3/week	7 (0.8)	1 (0.3)	6 (1.2)
None	829 (93.0)	360 (97.6)	469 (89.9)

*Asthma severity classification*^a^			
Intermittent	248 (26.5)	239 (62.4)	9 (1.6)
Mild persistent	237 (25.3)	75 (19.6)	162 (29.3)
Moderate persistent	302 (32.2)	65 (17.0)	237 (42.9)
Severe persistent	149 (15.9)	4 (1.0)	145 (26.2)

a Asthma severity classification is based on GINA guidelines in 2006. Results tabulated as mean (s.d.) or frequency (%).

**Table 2:** Univariate and multivariable analysis of primary outcome at baseline

	*Univariate model*		*Multivariable model*	
	*Odds ratio (95% CI)*	P *value*	*Odds ratio (95% CI)*	P *value*
Age (per year)	0.99 (0.99–1.00)	0.021	1.00 (0.99–1.00)	0.113

*Race*				
Malay versus Chinese	1.38 (0.98–1.94)	0.265	1.36 (0.95–1.94)	0.095
Indian versus Chinese	1.08 (0.74–1.60)	0.696	1.06 (0.71–1.57)	0.693
Others versus Chinese	1.94 (1.07–3.72)	0.035	1.93 (1.04–3.74)	0.042

*Written action plan*				
Been on plan versus No	0.49 (0.35–0.68)	<0.001	0.49 (0.35–0.68)	<0.001
Updated versus No	0.38 (0.25–0.57)	<0.001	0.37 (0.25–0.56)	<0.001

*Cigarette smoking*				
Ex versus Current	0.75 (0.38–1.44)	0.385	0.74 (0.37–1.45)	0.384
Never versus Current	0.63 (0.36–1.07)	0.098	0.60 (0.34–1.04)	0.076
Allergic Rhinitis (Yes versus No)	1.03 (0.78–1.37)	0.845		
Allergic Conjunctivitis (Yes versus No)	0.79 (0.37–1.70)	0.535		
Eczema (Yes versus No)	1.28 (0.80–2.10)	0.317		
Gender (Male versus Female)	1.10 (0.85–1.44)	0.460		

*Smoke as a trigger*				
Yes versus No	1.00 (0.36–3.03)	0.993		
Unknown versus No	0.93 (0.71–1.21)	0.581		

Only those variables significant in the univariate analysis at 0.1 were included in the multivariable analysis.

Abbreviation: CI, confidence interval.

**Table 3:** Longitudinal analysis of nocturnal symptoms as primary outcome

	*Univariate model*		*Multivariable model*	
	*Odds ratio (95% CI)*	P *value 9*	*Odds ratio (95% CI)*	P *value*
Age (per year)	0.993 (0.990–0.997)	<0.001	0.996 (0.993–0.999)	0.041

*Race*				
Malay versus Chinese	1.20 (0.96–1.49)	0.092	1.15 (0.93–1.42)	0.206
Indian versus Chinese	1.18 (0.92–1.51)	0.189	1.16 (0.91–1.48)	0.225
Others versus Chinese	1.35 (0.96–1.91)	0.084	1.31 (0.93–1.83)	0.122
Allergic rhinitis (Yes versus No)	1.51 (1.33–1.72)	<0.001	1.52 (1.34–1.73)	<0.001
Allergic conjunctivitis (Yes versus No)	1.65 (1.01–2.69)	0.045	1.30 (0.80–2.11)	0.288
Current visit (relative to last visit)	0.91 (0.89–0.93)	<0.001	0.91 (0.89–0.93)	<0.001
Eczema (Yes versus No)	1.10 (0.86–1.42)	0.440	—	—
Gender (Male versus Female)	1.04 (0.88–1.22)	0.648	—	—

*Smoke as a trigger*				
Yes versus No	1.02 (0.87–1.21)	0.778	—	—
Unknown versus No	0.93 (0.45–1.93)	0.855	—	—

This analysis has been adjusted for by visit within subject as a random effect and site as an independent random effect. Therefore, the odds ratio (OR) and the corresponding CI are conditional on the random effects. Only those variables significant in the univariate analysis at 0.1 were included in the multivariable analysis.

Abbreviation: CI, confidence interval.

**Table 4:** Longitudinal analysis of change in medication as secondary outcome

	*Univariate model*		*Multivariable model*	
	*Odds ratio (95% CI)*	P *value*	*Odds ratio (95% CI)*	P *value*
Nocturnal symptoms	2.63 (1.74–3.97)	<0.001	2.87 (1.88–4.38)	<0.001
Age (per year)	1.01 (1.00–1.17)	0.038	1.01 (1.00–1.02)	0.007
Period of medication usage (per month)	1.01 (1.00–1.02)	0.217		
Gender (Male versus Female)	0.97 (0.65–1.44)	0.86	—	—

*Race*				
Malay versus Chinese	0.78 (0.47–1.30)	0.34	—	—
Indian versus Chinese	0.86 (0.47–1.58)	0.63	—	—
Others versus Chinese	1.21 (0.46–3.19)	0.38	—	—
Allergic rhinitis (Yes versus No)	0.95 (0.61–1.48)	0.38	—	—
Allergic conjunctivitis (Yes versus No)	1.51 (0.14–16.7)	0.73	—	—
Eczema (Yes versus No)	0.74 (0.29–1.91)	0.53	—	—
Current visit (relative to last visit)	1.03 (0.99–1.07)	0.12	—	—

This analysis has been adjusted for by subject as a random effect.

Abbreviation: CI, confidence interval.
